# Impact of onset timing and operative duration on outcomes in chronic subdural hematoma: an exploratory retrospective study

**DOI:** 10.3389/fneur.2026.1741337

**Published:** 2026-05-07

**Authors:** Liang Wang, Jinjin Wang, Qingpeng Meng

**Affiliations:** Department of Neurosurgery, The Affiliated Chuzhou Hospital of Anhui Medical University (Chuzhou First People's Hospital), Chuzhou, Anhui, China

**Keywords:** chronic subdural haematoma, onset timing, prognosis, recurrence, symptom-to-surgery

## Abstract

**Objective:**

To investigate how onset timing and operative duration influence recurrence, efficacy, and postoperative recovery in patients with chronic subdural hematoma (CSDH).

**Methods:**

We conducted a retrospective analysis of 122 CSDH patients who underwent surgical treatment at the Department of Neurological Surgery, First People’s Hospital of Chuzhou City (Affiliated to Anhui Medical University) between January 2016 and October 2024. We collected data on demographics, clinical characteristics, surgical details, and outcome measures including Glasgow Coma Scale (GCS), modified Rankin Scale (mRS), Karnofsky Performance Status (KPS), and Eastern Cooperative Oncology Group (ECOG). Given the exploratory nature of this study and sample size constraints, we employed univariate analyses (independent t-tests, Mann–Whitney U tests, and chi-square tests) to compare outcomes between groups based on onset timing and operative duration, with Spearman correlation analysis used to examine relationships between these factors and prognostic indicators.

**Results:**

Patients with operative duration >60 min (*n* = 99) had significantly higher postoperative GCS and KPS scores than those with ≤60 min (*p* = 0.022 and 0.031, respectively). No significant differences were observed between the two groups in postoperative mRS (*p* = 0.149), ECOG score (*p* = 0.077), recurrence rate (*p* = 0.200), or complications (*p* = 0.605). Correlation analysis showed that operative duration (as a continuous variable) was weakly negatively correlated with postoperative mRS (*ρ* = −0.197, *p* = 0.030) and postoperative ECOG (*ρ* = −0.224, *p* = 0.013), and weakly positively correlated with postoperative KPS (*ρ* = 0.258, *p* = 0.004) and postoperative GCS (*ρ* = 0.263, p = 0.004). Symptom-to-surgery interval was associated only with longer hospital stay (*p* < 0.05); statin use showed no significant correlations with any outcome.

**Conclusion:**

In this exploratory analysis, operative duration >60 min was associated with better postoperative GCS and KPS scores in group comparisons, and weak but significant correlations were observed between operative duration and all four functional scales. These associations are likely confounded by baseline differences in patient status and surgical technique. Symptom-to-surgery interval and statin use did not significantly influence short-term prognosis. Larger prospective studies with multivariate adjustment are needed to confirm these preliminary findings.

## Introduction

Chronic subdural haematoma (CSDH) is a chronic occupying lesion that forms between the arachnoid and dura mater (1). It typically develops 3 weeks after head trauma, with an annual incidence of 1.7–20.6 per 100,000 cases, showing an increasing trend over time (2, 3). While CSDH commonly results from mild head trauma, its clinical presentation and prognosis exhibit considerable variation. The clinical course comprises three distinct periods: the initial traumatic event, the latent period, and the period of clinical manifestation (4). Although surgery remains the primary treatment modality for CSDH (5), patients may experience poor outcomes following haematoma removal. These include recurrence, postoperative complications (such as hemorrhage, infection, and epilepsy), and decreased ability to perform daily activities (6, 7). Despite recent advancements in surgical techniques and perioperative management, CSDH continues to present significant challenges, with high rates of postoperative recurrence and complications (8). This situation creates a substantial burden for patients, particularly regarding haematoma recurrence and unfavorable clinical outcomes.

The impact of presentation timing on prognosis remains debatable. While some research indicates early surgery may lead to better outcomes, other studies show minimal differences between early and delayed surgical interventions (9). Evidence suggests prolonged symptom duration correlates with decreased improvement rates (10), and anticoagulation therapy influences reoperation frequencies (11). The relationship between surgery duration and outcomes also requires further investigation (12). Extended surgical procedures may elevate postoperative complication risks, though specific temporal thresholds and underlying mechanisms remain undefined (13, 14). Notably, preoperative administration of statins or corticosteroids has effectively reduced haematoma evacuation failure rates (15). Furthermore, while existing research primarily focuses on individual predictive factors for CSDH prognosis – such as Jensen et al.’s (16) identification of radiological subtype, midline shift, haematoma volume, and postoperative drainage volume as significant factors – comprehensive multivariate analyses are limited. This gap is particularly significant given that many factors cannot be evaluated during the perioperative period, highlighting the need for an integrated approach to prognostic assessment.

Middle meningeal artery embolization with liquid embolic agents has emerged as a safe and effective therapeutic option for patients who have failed previous surgical interventions, serving as a viable alternative to conventional treatment (17). However, the risk of haematoma recurrence remains a concern with this procedure (18). This study aims to investigate how the timing of onset and surgical duration influence the prognosis of CSDH through comprehensive univariate and correlation analyses, providing preliminary evidence for clinical management. Given the sample size constraints, we focused on identifying potential associations rather than establishing independent predictors through multivariate modeling. Our investigation focuses on two key aspects: first, examining how the preoperative course duration affects prognostic indicators, including recurrence rates, GCS scores, mRS, KPS, and ECOG score changes; second, analyzing the relationship between surgical duration and postoperative outcomes. We hypothesize that earlier surgical intervention and shorter operative times will lead to significantly better prognoses for CSDH patients, with lower risks of poor outcomes and haematoma recurrence. Through this research, we aim to develop a more precise prognostic assessment tool to help clinicians optimize surgical strategies and enhance patient outcomes and quality of life.

## Information and methods

### Study design and population

We conducted a retrospective analysis of 122 patients with a first diagnosis of chronic subdural hematoma (CSDH) who underwent surgical treatment at the Department of Neurosurgery, First People‘s Hospital of Chuzhou City (Affiliated to Anhui Medical University) between January 1, 2016 and October 10, 2024. Inclusion criteria were: (1) initial diagnosis of CSDH; (2) discharge during the study period; (3) surgical intervention (drilling drainage, endoscopic surgery, or middle meningeal artery embolization). Exclusion criteria were: (1) previous craniotomy; (2) concurrent extracranial surgery; (3) postoperative sedation >6 h; (4) absent surgical indication (midline shift <0.5 cm without compression); (5) similar surgery within 6 months; (6) incomplete or unreliable surgical records.

### Variables and definitions

Onset time was defined as the interval from the first reported symptom (based on patient or family recall) to hospital admission. Operative duration was recorded as the time from skin incision to closure. Surgical techniques were categorized as trepanation and drainage, endoscopic surgery, MMA embolization, or craniotomy with bone flap. Postoperative statin use was defined as oral statin therapy initiated after surgery. Recurrence was defined as re-accumulation of hematoma with symptoms within 6 months (19).

### Outcome measures

Functional status was assessed using the GCS, mRS, KPS, and ECOG performance status. All scores were recorded at admission (preoperative) and at discharge (postoperative). Higher mRS and ECOG scores indicate worse disability; higher KPS and GCS scores indicate better function. Treatment efficacy was categorized as “cured” (complete symptom resolution and hematoma clearance), “improved” (partial symptom improvement with residual hematoma), or “ineffective” (no improvement).

### Statistical methods

Data were entered into Excel and analyzed using MedCalc software version 20.217 (64-bit). Continuous variables were tested for normality using the Shapiro–Wilk test. Normally distributed data were expressed as mean ± standard deviation and compared using independent samples *t*-tests. Non-normally distributed data were expressed as median (interquartile range) and compared using Mann–Whitney *U* tests. Categorical variables were presented as frequencies (percentages) and compared using chi-square tests or Fisher’s exact tests when appropriate. Given the ordinal nature of functional outcome scores, Spearman rank correlation was used for all correlation analyses. A two-tailed *p*-value < 0.05 was considered statistically significant. Graphs were created using GraphPad Prism 10 software. Given the sample size and the exploratory hypothesis-generating nature of this study, we opted for univariate analyses to identify potential associations. We acknowledge that this approach does not allow for adjustment of confounding factors. Therefore, the findings should be interpreted as preliminary and hypothesis-generating, rather than confirmatory. Missing data were handled by listwise deletion, as the proportion of missing values was minimal (e.g., <3% for key variables).

## Results

### General information of patients

A total of 122 patients were included. Baseline characteristics are summarized in Table 1. The median age was 70 years (IQR 64–79), and 86.9% were male. Median onset time was 7 days (IQR 3–15), and median operative duration was 86.5 min (IQR 60–120). Trepanation and drainage was the most common surgical technique (82.8%). Preoperative functional scores varied widely, with 66.7% of patients having a preoperative GCS of 15 and 37.5% having a preoperative mRS of 4. Postoperatively, 87.7% were cured, and recurrence occurred in 6.6%.

**Table 1:** Baseline characteristics of the study population (*N* = 122).

Characteristic	Value
Age, years, median (IQR)	70 (64–79)
Sex, *n* (%)	
Male	106 (86.9)
Female	16 (13.1)
Onset time, days, median (IQR)	7 (3–15)
Operative duration, min, median (IQR)	86.5 (60–120)
Length of hospital stay, days, median (IQR)	17.5 (12–19)
Statin use, *n* (%)	64 (52.5)
Surgical technique, *n* (%)	
Trepanation and drainage	101 (82.8)
Endoscopic surgery	14 (11.5)
MMA embolization	4 (3.3)
Craniotomy with bone flap	3 (2.5)
Hematoma side, *n* (%)^a^	
Left	58 (48.7)
Right	30 (25.2)
Bilateral	31 (26.1)
Missing	3
Preoperative GCS, *n*^b^	
8	1 (0.8)
9	4 (3.3)
10	1 (0.8)
11	3 (2.5)
12	9 (7.5)
13	6 (5.0)
14	16 (13.3)
15	80 (66.7)
Preoperative mRS, *n*^b^	
1	38 (31.7)
2	17 (14.2)
3	14 (11.7)
4	45 (37.5)
5	6 (5.0)
Preoperative KPS, *n*^b^	
20	7 (5.8)
30	13 (10.8)
40	24 (20.0)
50	8 (6.7)
60	1 (0.8)
70	9 (7.5)
80	21 (17.5)
90	37 (30.8)
Preoperative ECOG, *n*^b^	
1	48 (40.0)
2	16 (13.3)
3	32 (26.7)
4	24 (20.0)
Postoperative outcomes	
Recurrence, *n* (%)	8 (6.6)
Postoperative complications, *n* (%)	5 (4.1)
Efficacy, *n* (%)	
Cured	107 (87.7)
Improved	10 (8.2)
No effect	5 (4.1)

^a^Hematoma side had 3 missing cases; percentages calculated among 119 patients with available data.

^b^Preoperative scores had 2 missing cases; percentages calculated among 120 patients with complete data.

### Comparison of preoperative conditions of patients with different onset times

Comparing the gender, age, type of surgery and preoperative mRS, KPS, ECOG score, GCS score level of patients in the onset <30d group and the ≥30d group, the differences were not statistically significant (*p* > 0.05) (Table 2).

**Table 2:** Comparison of preoperative conditions of patients with different onset times.

Variable	Overall, *N* = 122	Onset times ≤ 30d, *N* = 106	Onset times>30d, *N* = 16	*Χ*^2^/*Z*	*p*-value
Sex				0.226	0.634
Male	106 (87%)	91 (86%)	15 (94%)		
Female	16 (13%)	15 (14%)	1 (6.2%)		
Age (years)	70 (64, 79)	70 (63, 79)	70 (66, 77)	−0.061	0.952
Preoperative GCS score				4.688	0.698
8	1 (0.8%)	1 (1.0%)	0 (0%)		
9	4 (3.3%)	4 (3.8%)	0 (0%)		
10	1 (0.8%)	1 (1.0%)	0 (0%)		
11	3 (2.5%)	2 (1.9%)	1 (6.2%)		
12	9 (7.5%)	9 (8.7%)	0 (0%)		
13	6 (5.0%)	5 (4.8%)	1 (6.2%)		
14	16 (13%)	15 (14%)	1 (6.2%)		
15	80 (67%)	67 (64%)	13 (81%)		
NA	2	2	0		
mRS (preoperative)				1.599	0.809
1	38 (32%)	32 (31%)	6 (38%)		
2	17 (14%)	14 (13%)	3 (19%)		
3	14 (12%)	12 (12%)	2 (12%)		
4	45 (38%)	40 (38%)	5 (31%)		
5	6 (5.0%)	6 (5.8%)	0 (0%)		
NA	2	2	0		
KPS (preoperative)				8.853	0.205
20	7 (5.8%)	7 (6.7%)	0 (0%)		
30	13 (11%)	10 (9.6%)	3 (19%)		
40	24 (20%)	24 (23%)	0 (0%)		
50	8 (6.7%)	7 (6.7%)	1 (6.2%)		
60	1 (0.8%)	1 (1.0%)	0 (0%)		
70	9 (7.5%)	7 (6.7%)	2 (12%)		
80	21 (18%)	18 (17%)	3 (19%)		
90	37 (31%)	30 (29%)	7 (44%)		
NA	2	2	0		
ECOG score (preoperative)				2.181	0.568
1	48 (40%)	39 (38%)	9 (56%)		
2	16 (13%)	14 (13%)	2 (12%)		
3	32 (27%)	29 (28%)	3 (19%)		
4	24 (20%)	22 (21%)	2 (12%)		
NA	2	2	0		
Application of statins	64 (52%)	58 (55%)	6 (38%)	1.652	0.199
Surgical site				6.386	0.172
Left	53 (48%)	50 (53%)	3 (20%)		
Right	32 (29%)	25 (26%)	7 (47%)		
Bilateral	25 (23%)	20 (21%)	5 (33%)		
NA	12	11	1		
Type of surgery				2.168	0.538
MMA Embolization	4 (3.3%)	4 (3.8%)	0 (0%)		
Craniotomy with bone flap	3 (2.5%)	2 (1.9%)	1 (6.2%)		
Endoscopic Surgery	14 (11%)	13 (12%)	1 (6.2%)		
Trepanation and Drainage	101 (83%)	87 (82%)	14 (88%)		

Data presented as *n* (%) or median (IQR). *p*-values from Fisher’s exact test, Wilcoxon rank sum test, or Pearson’s chi-squared test as appropriate.

### Comparison of preoperative conditions of patients with different operation times

Comparing the gender, age, type of surgery and preoperative mRS, KPS, ECOG score between the operative duration ≤60 min group and the >60 min group, the differences were not statistically significant (*p* > 0.05). However, patients in the >60 min group had significantly higher preoperative GCS scores and a higher proportion of bilateral surgeries (*p* < 0.05) (Table 3).

**Table 3:** Comparison of preoperative conditions of patients with different operative durations.

Variable	Overall, *N* = 122	Operation time ≤ 60 min, *N* = 23	Operation time > 60 min, *N* = 99	*Χ*^2^/*Z*	*p*-value^2^
Sex				4.186	0.078
Male	106 (87%)	17 (74%)	89 (90%)		
Female	16 (13%)	6 (26%)	10 (10%)		
Age	70 (64, 79)	78 (66, 83)	69 (64, 77)	0.061	0.952
Preoperative GCS score				17.346	0.015
8	1 (0.8%)	1 (4.3%)	0 (0%)		
9	4 (3.3%)	1 (4.3%)	3 (3.1%)		
10	1 (0.8%)	1 (4.3%)	0 (0%)		
11	3 (2.5%)	2 (8.7%)	1 (1.0%)		
12	9 (7.5%)	3 (13%)	6 (6.2%)		
13	6 (5.0%)	0 (0%)	6 (6.2%)		
14	16 (13%)	4 (17%)	12 (12%)		
15	80 (67%)	11 (48%)	69 (71%)		
NA	2	0	2		
mRS (preoperative)				7.416	0.115
1	38 (32%)	6 (26%)	32 (33%)		
2	17 (14%)	2 (8.7%)	15 (15%)		
3	14 (12%)	5 (22%)	9 (9.3%)		
4	45 (38%)	7 (30%)	38 (39%)		
5	6 (5.0%)	3 (13%)	3 (3.1%)		
NA	2	0	2		
KPS (preoperative)				3.490	0.836
20	7 (5.8%)	3 (13%)	4 (4.1%)		
30	13 (11%)	3 (13%)	10 (10%)		
40	24 (20%)	4 (17%)	20 (21%)		
50	8 (6.7%)	1 (4.3%)	7 (7.2%)		
60	1 (0.8%)	0 (0%)	1 (1.0%)		
70	9 (7.5%)	2 (8.7%)	7 (7.2%)		
80	21 (18%)	4 (17%)	17 (18%)		
90	37 (31%)	6 (26%)	31 (32%)		
NA	2	0	2		
ECOG score (preoperative)				3.012	0.390
1	48 (40%)	8 (35%)	40 (41%)		
2	16 (13%)	4 (17%)	12 (12%)		
3	32 (27%)	4 (17%)	28 (29%)		
4	24 (20%)	7 (30%)	17 (18%)		
NA	2	0	2		
Application of statins	64 (52%)	15 (65%)	49 (49%)	1.850	0.174
Surgical site				9.720	0.045
Left	53 (48%)	14 (64%)	39 (44%)		
Right	32 (29%)	8 (36%)	24 (27%)		
Bilateral	25 (23%)	0 (0%)	25 (28%)		
NA	12	1	11		
Type of surgery				10.259	0.114
MMA Embolization	4 (3.3%)	0 (0%)	4 (4.0%)		
Craniotomy with bone flap	3 (2.5%)	0 (0%)	3 (3.0%)		
Endoscopic Surgery	14 (11%)	0 (0%)	14 (14%)		
Trepanation and Drainage	101 (83%)	23 (100%)	78 (79%)		

Data presented as *n* (%) or median (IQR). *P*-values from Fisher’s exact test, Wilcoxon rank sum test, or Pearson’s chi-squared test as appropriate.

### Comparison of postoperative conditions of patients with different onset times

Comparing efficacy, recurrence rate, and postoperative mRS, KPS, ECOG, and GCS scores between patients with different onset times, the differences were not statistically significant (*p* > 0.05). Length of stay was significantly longer for those with disease duration >30 days (*p* < 0.05) (Table 4).

**Table 4:** Comparison of postoperative conditions of patients with different onset times.

Variable	Overall, *N* = 122	Onset times < 30d, *N* = 106	Onset times ≥ 30d, *N* = 16	*Χ*^2^/*Z*	*p*-value^2^
Efficacy				0.618	0.432
No effect	5 (4.1%)	4 (3.8%)	1 (6.2%)		
Improved	10 (8.2%)	8 (7.5%)	2 (12%)		
Cured	107 (88%)	94 (89%)	13 (81%)		
Postoperative GCS score				0.693	0.405
10	1 (0.8%)	1 (1.0%)	0 (0%)		
11	3 (2.5%)	2 (1.9%)	1 (6.2%)		
15	116 (97%)	101 (97%)	15 (94%)		
NA	2	2	0		
mRS (postoperative)				0.693	0.405
0	85 (71%)	71 (69%)	14 (88%)		
1	21 (18%)	20 (19%)	1 (6.2%)		
2	4 (3.4%)	4 (3.9%)	0 (0%)		
3	5 (4.2%)	5 (4.9%)	0 (0%)		
4	4 (3.4%)	3 (2.9%)	1 (6.2%)		
NA	3	3	0		
Postoperative complication	5 (4.1%)	5 (4.7%)	0 (0%)	0.044	0.833
KPS (postoperative)				11.354	0.124
30	1 (0.8%)	0 (0%)	1 (6.2%)		
40	2 (1.7%)	2 (1.9%)	0 (0%)		
50	3 (2.5%)	3 (2.9%)	0 (0%)		
60	1 (0.8%)	1 (1.0%)	0 (0%)		
70	1 (0.8%)	1 (1.0%)	0 (0%)		
80	6 (5.0%)	5 (4.9%)	1 (6.2%)		
90	20 (17%)	20 (19%)	0 (0%)		
100	85 (71%)	71 (69%)	14 (88%)		
NA	3	3	0		
ECOG score (postoperative)				6.694	0.105
0	85 (72%)	71 (70%)	14 (88%)		
1	20 (17%)	20 (20%)	0 (0%)		
2	7 (5.9%)	6 (5.9%)	1 (6.2%)		
3	4 (3.4%)	4 (3.9%)	0 (0%)		
4	2 (1.7%)	1 (1.0%)	1 (6.2%)		
NA	4	4	0		
Recurrence	8 (6.6%)	7 (6.6%)	1 (6.2%)	0.000	1.000
Length of stay (days)	17.5 (12, 19)	15 (12, 18)	18 (12.75, 22)	2.002	0.045

Data presented as n (%) or median (IQR). *P*-values from Fisher’s exact test, Wilcoxon rank sum test, or Pearson’s chi-squared test as appropriate.

### Comparison of postoperative conditions of patients with different operation times

Patients with operative duration >60 min had significantly higher postoperative GCS scores (*p* = 0.022) and KPS (*p* = 0.031) compared to those with ≤60 min. Postoperative mRS, ECOG scores, recurrence rates, and complications showed no statistically significant differences between groups (*p* > 0.05) (Table 5).

**Table 5:** Comparison of prognosis of patients with different operation times.

Variable	Overall, *N* = 122	Operation time ≤ 60 min, *N* = 23	Operation time > 60 min, *N* = 99	*Χ*^2^/*Z*	*p*-value^2^
Efficacy				1.878	0.391
No effect	5 (4.1%)	0 (0%)	5 (5.1%)		
Improved	10 (8.2%)	1 (4.3%)	9 (9.1%)		
Cured	107 (88%)	22 (96%)	85 (86%)		
Postoperative GCS score				8.864	0.022
10	1 (0.8%)	1 (4.3%)	0 (0%)		
11	3 (2.5%)	2 (8.7%)	1 (1.0%)		
15	116 (97%)	20 (87%)	96 (99%)		
NA	2	0	2		
mRS (postoperative)				5.795	0.149
0	85 (71%)	13 (57%)	72 (75%)		
1	21 (18%)	5 (22%)	16 (17%)		
2	4 (3.4%)	1 (4.3%)	3 (3.1%)		
3	5 (4.2%)	2 (8.7%)	3 (3.1%)		
4	4 (3.4%)	2 (8.7%)	2 (2.1%)		
NA	3	0	3		
Postoperative complication	5 (4.1%)	0 (0%)	5 (5.1%)	0.267	0.605
KPS (postoperative)				13.368	0.031
30	1 (0.8%)	1 (4.3%)	0 (0%)		
40	2 (1.7%)	1 (4.3%)	1 (1.0%)		
50	3 (2.5%)	1 (4.3%)	2 (2.1%)		
60	1 (0.8%)	1 (4.3%)	0 (0%)		
70	1 (0.8%)	1 (4.3%)	0 (0%)		
80	6 (5.0%)	1 (4.3%)	5 (5.2%)		
90	20 (17%)	4 (17%)	16 (17%)		
100	85 (71%)	13 (57%)	72 (75%)		
NA	3	0	3		
ECOG score (postoperative)				7.487	0.077
0	85 (72%)	13 (57%)	72 (76%)		
1	20 (17%)	4 (17%)	16 (17%)		
2	7 (5.9%)	3 (13%)	4 (4.2%)		
3	4 (3.4%)	2 (8.7%)	2 (2.1%)		
4	2 (1.7%)	1 (4.3%)	1 (1.1%)		
NA	4	0	4		
Recurrence	8 (6.6%)	3 (13%)	5 (5.1%)	1.645	0.200
Length of stay (days)	17.5 (12, 19)	16 (11.25, 17.00)	15 (12, 19.75)	0.246	0.806

Data presented as *n* (%) or median (IQR). *P*-values from Fisher’s exact test, Wilcoxon rank sum test, or Pearson’s chi-squared test as appropriate.

### Correlation analysis of time to surgery, time to onset and patient prognosis-related indicators

**Figure 1:**
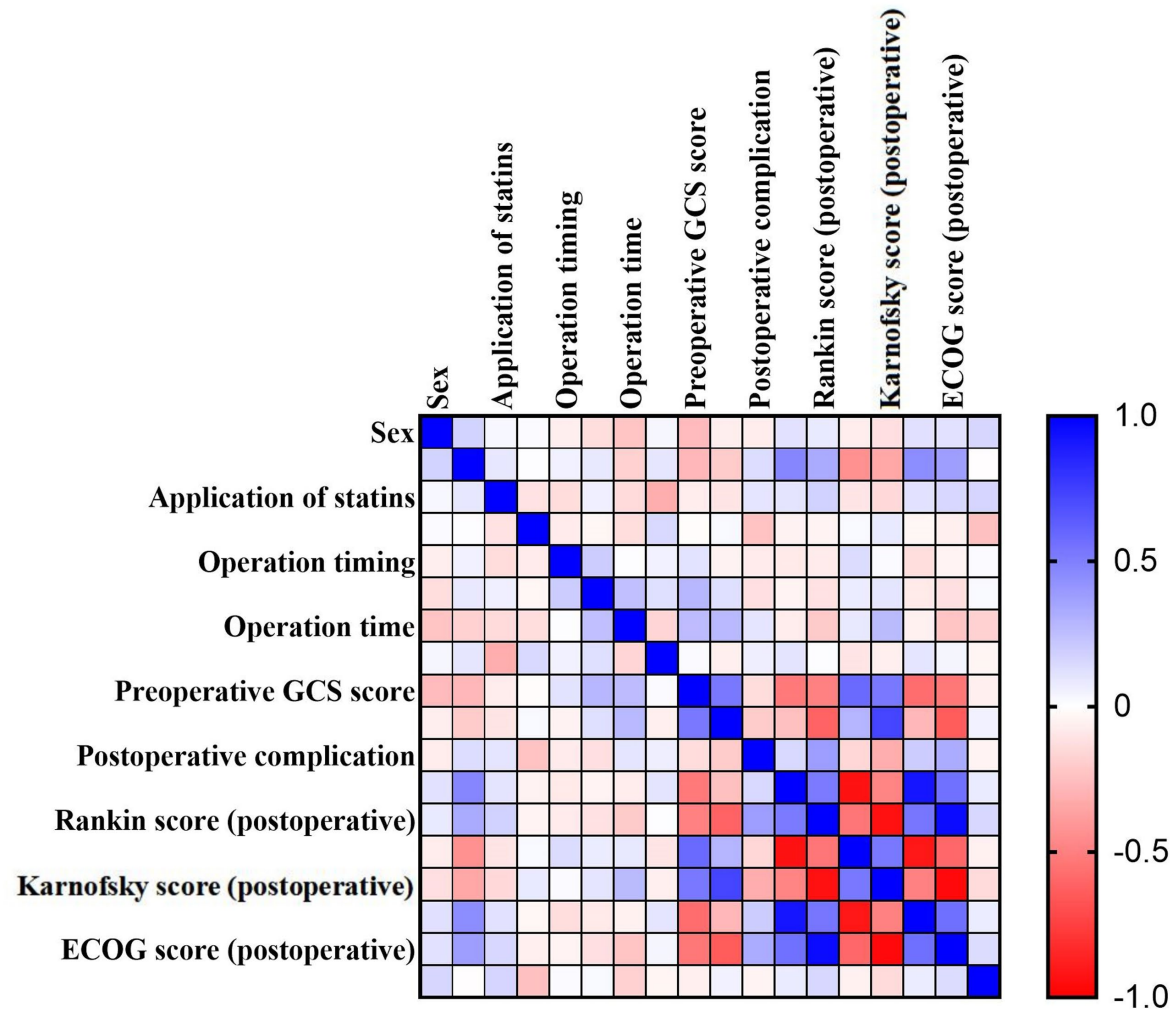
Heat map of the correlation of each indicator.

No significant correlations were found between onset timing or statin use and any prognostic indicator. Operative duration showed weak negative correlations with postoperative mRS (*ρ* = −0.197, *p* = 0.030), postoperative ECOG score (*ρ* = −0.224, *p* = 0.013), and efficacy (*ρ* = −0.118, *p* = 0.195), and weak positive correlations with postoperative KPS (*ρ* = 0.258, *p* = 0.004) and postoperative GCS score (*ρ* = 0.263, *p* = 0.004) (Table 6 and Figure 1).

**Table 6:** Spearman correlation between preoperative indicators and patient prognosis.

Variable	Efficacy	Postoperative complications	mRS (postoperative)	KPS (postoperative)	ECOG score (postoperative)	Recurrence	Postoperative GCS
Age	0.011	0.117	0.322**	−0.336**	0.376**	−0.026	−0.200*
Onset times	−0.071	−0.08	−0.077	0.015	−0.037	−0.005	−0.055
Operation time	−0.118	0.100	−0.197*	0.258**	−0.224*	−0.126	0.263**
Type of surgery	0.099	0.019	0.027	−0.089	0.067	0.005	−0.074
Preoperative GCS	−0.005	−0.098	−0.486**	0.528**	−0.533**	−0.069	0.513**
mRS (Preoperative)	−0.058	0.076	0.513**	−0.485**	0.549**	0.078	−0.238**
KPS (Preoperative)	0.035	−0.094	−0.522**	0.521**	−0.582**	−0.061	0.281**
ECOG (preoperative)	−0.049	0.129	0.543**	−0.502**	0.562**	0.078	−0.271**
Applying statins	−0.088	0.114	0.141	−0.103	0.119	0.12	−0.089
Sex	0.032	−0.08	0.048	−0.094	0.075	0.093	−0.055

*P < 0.05, **p < 0.01. Spearman correlation coefficients are reported. Operation time showed weak, non-significant correlation with efficacy (*ρ* = −0.118, *P* = 0.195).

## Discussion

In this exploratory retrospective study of 122 CSDH patients, we found that patients with operative duration >60 min had significantly higher postoperative GCS and KPS scores compared to those with shorter operative duration. However, no significant differences were observed between the two groups in postoperative mRS, ECOG, recurrence rate, or complications. Correlation analysis revealed that operative duration (as a continuous variable) was weakly but significantly correlated with all four functional outcome measures (mRS, ECOG, KPS, and GCS). These findings initially appear counterintuitive, as longer operative time might be expected to worsen outcomes, but they likely reflect confounding by baseline patient status and surgical technique rather than a direct protective effect of prolonged surgery. These preliminary observations highlight the complexity of CSDH prognosis and the need for cautious interpretation (14, 20).

The apparent association between longer operative time and better functional recovery is likely driven by confounding factors rather than a direct protective effect of prolonged surgery. First, as shown in Table 3, patients in the longer operative duration group had significantly higher preoperative GCS scores (*p* = 0.015), indicating less severe neurological impairment at baseline—a factor that could predispose them to better recovery regardless of operative duration. Second, all patients in the longer-duration group underwent trepanation and drainage, whereas the shorter-duration group included a mix of techniques (endoscopic surgery, MMA embolization, craniotomy). These procedures vary intrinsically in complexity and operative time: endoscopic surgery may reduce operative time compared to craniotomy (21), and the subdural evacuating port system (SEPS) has been associated with shorter operative times (22). Furthermore, the longer-duration group had a significantly higher proportion of bilateral surgeries (*p* = 0.045), which inherently requires more operative time and may reflect more complex pathology (23). Thus, the operative duration in our study may serve as a surrogate for surgical technique and baseline patient status rather than an independent prognostic factor (24, 25). This interpretation is consistent with recent network meta-analyses showing that different surgical techniques for CSDH have comparable efficacy but varying operative times and complication profiles (14).

Our finding that onset timing was not associated with postoperative outcomes contrasts with some previous reports. For instance, Ro et al. (19) identified short symptom-to-surgery duration as a predictor of good brain re-expansion and lower recurrence. The discrepancy may stem from differences in outcome definitions, study populations, or the inherent limitations of retrospectively collected onset data—particularly in our cohort where 33% of patients had preoperative GCS ≤ 14, making reliable symptom recall challenging. Similarly, we found no significant correlation between statin use and prognosis, despite prior suggestions that preoperative statins might reduce hematoma evacuation failure (15). This negative finding could reflect the observational nature of our study (with potential indication bias) or the fact that statin use was postoperative rather than preoperative. Other established prognostic factors, such as hematoma volume, midline shift, and drainage volume (16), were not assessed in our univariate framework, and their absence may have obscured true associations. Collectively, these results emphasize the need for prospective studies with standardized data collection to disentangle the effects of time-related and pharmacological variables.

Several important limitations must be acknowledged. First, the retrospective design introduces inherent selection and information biases, and unmeasured confounding cannot be excluded. Second, due to the modest sample size (*N* = 122) and exploratory nature, we did not perform multivariate regression analysis; thus, we could not adjust for key confounders such as preoperative neurological status, hematoma characteristics, surgeon experience, or postoperative care protocols (16, 26). Third, the heterogeneity of surgical techniques (trepanation and drainage, endoscopic surgery, MMA embolization, craniotomy) is a major confounder, as operative time is inextricably linked to procedure type (21, 22, 24, 25). Fourth, onset time relied on patient or family recall, which is subject to recall bias—particularly in patients with impaired consciousness. Fifth, the sample size in subgroups (e.g., >60-min group, *n* = 99; <60-min group, *n* = 23) provided adequate power for primary comparisons, but some subgroup analyses (e.g., recurrence rate: 5.1% vs. 13%) may still be underpowered. Finally, single-center data may limit generalizability. These limitations caution against causal interpretation and position our findings as hypothesis-generating.

Despite these limitations, our exploratory analysis offers valuable insights for future research. The counterintuitive association between longer operative time and better outcomes highlights the critical need for multivariate approaches that account for baseline status and surgical case mix. Future studies should employ prospective, multicenter designs with larger cohorts, standardized protocols for data collection (including detailed radiological parameters (16, 27) and postoperative management), and advanced analytic methods such as multivariate regression or propensity score matching. The role of MMA embolization as a primary or adjunctive treatment (17, 18) and the potential benefits of pharmacotherapies like statins (15) require rigorous evaluation in randomized controlled trials. Ultimately, integrating patient factors, radiological findings, surgical variables, and postoperative events into comprehensive prediction models (16) may enable personalized prognostic assessment and guide clinical decision-making in CSDH.

## Conclusion

In this exploratory retrospective study, operative duration >60 min was associated with better postoperative GCS and KPS scores in group comparisons, and weak but significant correlations were observed between operative duration (as a continuous variable) and all four functional outcome measures. However, these associations are likely confounded by baseline differences in patient status and surgical technique. Symptom-to-surgery interval and statin use showed no significant associations with short-term prognosis. These hypothesis-generating findings underscore the need for larger, prospective studies with multivariate adjustment to identify independent predictors of CSDH prognosis and to clarify the role of time-related factors in clinical outcomes.

## Data Availability

The original contributions presented in the study are included in the article/supplementary material, further inquiries can be directed to the corresponding author.
